# Willingness to Share yet Maintain Influence: A Cross-Sectional Study on Attitudes in Sweden to the Use of Electronic Health Data

**DOI:** 10.1093/phe/phaa035

**Published:** 2020-11-27

**Authors:** Sara Belfrage, Niels Lynöe, Gert Helgesson

**Affiliations:** Stockholm Centre for Healthcare Ethics; Department of Learning, Informatics, Management and Ethics; Karolinska Institutet

## Abstract

We have investigated attitudes towards the use of health data among the Swedish population by analyzing data from a survey answered by 1645 persons. Health data are potentially useful for a variety of purposes. Yet information about health remains sensitive. A balance therefore has to be struck between these opposing considerations in a number of contexts. The attitudes among those whose data is concerned will influence the perceived legitimacy of policies regulating health data use. We aimed to investigate what views are held by the general public, and what aspects matter for the willingness to let one’s data be used not only for one’s own care but also for other purposes. We found that while there is a broad willingness to let one’s data be used, the possibility to influence that use is considered important. The study also indicated that when respondents are required to balance different interests, priority is typically given to compulsory schemes ensuring that data are available where needed, rather than voluntary participation and data protection. The policy implications to be drawn from this are not self-evident, however, since the fact that a majority has a certain attitude does not by itself determine the most adequate policy.

## Introduction

Electronic health data from medical records and registries covering specific patient groups or entire populations are used for an increasing number of purposes, to the benefit of the individual, future patients and society at large. Examples of beneficial use are well-coordinated and efficient care for the patient, infection tracing, quality measurement and improvement in healthcare, and medical research, increasingly often using big data (Nuffield Council on Bioethics, 2015; Organisation for Economic Co-operation and Development, OECD, 2015; Vårdanalys, 2016; Garvin *et al.*, 2020). There is no reason to believe that future benefits will decrease—on the contrary, with increased availability (e.g. mobile data) and rapid advancement of analytics techniques for use on big data, there is hope for more accurate, rich, relevant and timely information (Kalra and Fernando, 2013; Jiang *et al.*, 2017; Lee, 2017). At the same time, information about a person’s health is sensitive and its handling involves risks, primarily relating to privacy. If health data ends up in the wrong hands, this may cause e.g. economic, psychological and social harm, such as stigmatization (Rachels, 1975; Sunstein, 2001; Eriksson and Helgesson, 2005; Ponemon Institute, 2015; van den Hoven *et al.*, 2018). In a larger perspective, handling of health data also involves risks of harm to broader public interests due to, for instance, data breach or fear thereof (Lupton, 2012; Laurie *et al.*, 2014; Mittelstadt and Floridi, 2016; Blumenthal, 2017). In other words—the use of health data involves competing interests, and a balance must be struck when shaping the regulations for how health data may be used for different purposes.

For health data regulations to be perceived as legitimate, the general public must find the choices made regarding how this balance is struck acceptable (cf. Skovgaard *et al.* 2019). This concerns matters like for what purposes electronic health data may be used, whether individuals are granted the possibility to influence the collection and use of their data, and aspects concerning practical data handling and protection.

The present cross-sectional study focuses on a random sample of the population in Sweden, which constitutes an object of investigation for a number of reasons. Firstly, advanced information technology has for long formed a natural part of many people’s lives in Sweden, and the level of digital literacy in the population is high (Post- och telestyrelsen, 2017; Internetstiftelsen, 2018; European Commission, 2019). Secondly, digitalization was introduced early in the Swedish healthcare system compared to other countries. For instance, electronic medical records have existed for many years and health-related registers covering the whole population even longer (Rosén, 2002; Rynning, 2007; Kierkegaard, 2011). At the same time, there are weaknesses in the present healthcare system relating to the use of digital health data, primarily having to do with difficulties in exchanging electronic medical records between clinics, hospitals and other healthcare providers (Vårdanalys, 2016; The Economist Intelligence Unit, 2019). There are also restrictions on how health data may be collected in databases for research (Registerforskningsutredningen, 2014; Ludvigsson *et al.* 2016). This situation has resulted in a public debate marked by a polarization between those who point to the urgency of more unrestricted access to health data and those insisting on rigid data protection mechanisms (Vårdanalys, 2016). Also, other countries have experienced infected debates concerning the usage of health data, with the discussions around the nationwide health data project ‘*care.data*’ in the UK being a case in point (van Staa *et al.*, 2016; Skovgaard *et al.*, 2019).

In the case of Sweden, the lively debate has been paired with a lack of knowledge about the views of the population—hence motivating the work presented in this article.

There is some previous literature on the attitudes of the Swedish population towards the use of health data. Two Eurobarometers asked respondents whether providing personal information is a big issue to them and whether they would be willing to share personal data for different purposes (European Commission, 2015, 2019), and some studies on patients’ attitudes towards accessing their own medical records exist (e.g. Hägglund *et al.*, 2018; Moll *et al.*, 2018). Scott Duncan and Hägglund report a qualitative study on attitudes in Sweden towards the use of electronic health records in clinical trials (Scott Duncan and Hägglund, 2018), and Rynning reports results from a quantitative study (Rynning 2007). To our knowledge, recent comprehensive quantitative investigations about the attitudes in Sweden towards the use of health data more broadly are lacking. For instance, no data concerning Sweden were included in a recent review of investigations of attitudes towards the reuse of health data among people in the European Union (Skovgaard *et al.*, 2019). The present article aims at contributing to filling that empirical gap and explore the policy implications of the findings. More specifically, the following research questions are addressed:

- What acceptance does the population have for the use of their health data for different purposes?
- Does the population consider it important to be able to influence how one’s data is used?
- How important is the ability to influence the use of one’s health data relative to the importance of obtaining the benefits from data use?
- Does the population consider it important to avoid unauthorized access to one’s health data?
- How important is it to avoid unauthorized access to health data relative to the importance of obtaining the benefits from data being accessible?
- What policy implications are suggested by these findings?

### Study Population and Methods

This cross-sectional study is based on a questionnaire survey containing questions about attitudes towards the use of the respondents’ health data in primarily electronic medical records and registries. Results from the survey were published in a report in Swedish in 2017 (Vårdanalys, 2017).

### Sample and Recruitment of Participants

The target group was the Swedish population, 18 years old or above. The IDM Address register was used as sampling frame. The questionnaire was sent to 5460 persons, divided into 30 strata based on region of residence and age. Strata with an expected lower response rate were over-sampled with the aim to generate responses from a broad selection of the population and making comparisons based on age and region possible. However, in the present study, we have not investigated potential differences between different subgroups.

### The Questionnaire

The development of the questionnaire took its starting point in the literature and in interviews with patients and members of the public. It was further developed with the help of so-called cognitive interviews with 10 interviewees (Vårdanalys, 2017). The final questionnaire was comprised of 60 questions, out of which 3 allowed free-text answers and 13 concerned background variables. The questions included the following central themes: the individual’s access to her medical records, access to medical records within the healthcare system, the individual’s control over her medical records, the use of the medical records for other purposes than the individual’s own care, registries and databases where health data are collected and privacy risks of health data. There was no uniform format for the questions—some were straight forward while others involved weighing of interests by positioning one’s opinion on a scale between two extremes. In the present article, a subset of the questions from this questionnaire was used, relating to attitudes to the use of one’s health data, willingness to share health data, the need for data protection, use of routines for informed consent in relating to data use and how to balance conflicting interests in relation to the protection and use of personal health data (see Supplementary Appendix). The survey also contained a number of background questions, listed in Table 1.

**Table 1.** Descriptive statistics about respondents regarding sex, age, education, place of birth and health status. Results presented as proportions.

Sex	
Female (*n*% = 912)	56.1%
Male (*n* = 709)	43.5%
Others (*n* = 8)	0.4%
Age	
18–24 years (*n* = 278)	16.9%
25–34 years (*n* = 264)	16.0%
35–49 years (*n* = 386)	23.5%
50–64 years (*n* = 352)	21.4%
>65 years (*n* = 365)	22.2%
Education	
Primary school (*n* = 223)	13.7%
Secondary school (*n* = 677)	41.7%
University education (*n* = 725)	44.6%
Place of birth	
Sweden/Nordic countries (*n* = 1463)	89.6%
Europe (*n* = 65)	4.0%
Outside Europe (*n* = 104)	6.4%
Self-estimated health status	
Good or very good (*n* = 1235)	75.8%
Fair (*n* = 318)	19.5%
Bad or very bad (*n* = 77)	4.7%
Working within healthcare	
Yes (*n* = 316)	19.4%
No (*n* = 1312)	80.6%

### Data Collection

Data were collected by a postal questionnaire with the additional possibility to answer online. First, a notification was sent out providing potential respondents with the address to the online questionnaire, followed a week later by the printed questionnaire. Two reminders with the printed questionnaire were sent out. The survey was first distributed in December 2016 and closed in March 2017.

### Statistical Analysis

Most of the results are presented as proportions with a 95% confidence interval. Confidence intervals which are not overlapping might be understood as significant as if a hypothesis test had been conducted with a 0.05 per cent significance level. Answers such as ‘I don’t know’ and ‘I don’t have an opinion’ were treated as internal drop-outs and were excluded from calculations and analyses.

### Ethical Aspects

The questionnaire data were used following approval from the Regional Ethical Review Board (dnr 2018/872-31/5). The data used were pseudonymized, meaning that they cannot be attributed to specific data subjects without the use of additional information. This additional information—the ‘key’ revealing the identity of survey respondents—was not available to the researchers.

## Results

### Description of Respondents

The questionnaire was sent to 5460 individuals. One hundred and fifteen were returned to sender. 1645 persons responded—out of which 345 online—which means that the response rate was 30.8 per cent. Of those responding, a significantly larger proportion were women [56.1 per cent (CI 53.7–58.5) compared to 43.9 per cent (CI 41.5–46.3) for men]. The proportion of responders was somewhat lower for younger respondents (Table 1). The proportion of respondents not answering a particular question (the internal dropout rate) varied between 1 per cent and 9 per cent for the questions we analyzed.

### Broad Willingness to Allow Use of One’s Health Data for a Variety of Purposes

Of those stating a view on the matter (*n* = 1446), a large majority of respondents expressed a wish that their health data be available for uses relating to their own care; 94.4 per cent (CI 93.2–95.6) answered that they are ‘mostly in favor’ of healthcare units having access to patients’ electronic medical records from other healthcare units; 5.6 per cent (CI 4.4–6.8) were ‘mostly against’.

Respondents also expressed a positive attitude towards allowing information from their medical records to be used for other purposes than their own care (Table 2). A large majority was in favor of medical records being used for the purpose of medical follow-up within healthcare, certain research and education within healthcare.

**Table 2.** Willingness to allow authorized healthcare staff to use information from patients’ medical records for follow-up for quality assurance, certain research and clinical education

Should information in medical records be used by authorized staff for…			
	**… quality assurance?** (*n* = 1460)	**… certain research?** (*n* = 1452)	**… clinical education?** (*n* = 1405)
Yes, even without patient consent	46.8% (43.5–50.5)	35.9% (31.8–40.0)	29.1% (24.7–33.5)
Yes, but only with patient consent	50.5% (45.9–54.1)	61.0% (58.6–65.0)	66.3% (63.5–60.5)
No, never	2.7% (0–7.7)	3.1% (0–8.1)	4.6% (0–9.7)

Results presented as proportions with a 95% confidence interval.

### Desire to Maintain Influence over How One’s Health Data is Used

Table 2 also sheds light on whether the population considers it important to be able to influence the use of one’s health data. For all purposes taken a stand on, more respondents accepted the use of health data from medical records under the condition of patient consent, compared to use without patient consent.

That respondents accept their data to be used for a variety of reasons, but that the possibility to influence this use is important, is also shown in Table 3. It shows that a large majority (96 per cent) expressed their willingness to have information about their health included in health-related registers for research, follow-up and development, although under different conditions.

**Table 3.** Attitudes towards health data being entered into registers for research

What is your view on information about you being entered into such registries [used for research, follow-up and development]?	
I am willing to share my information and do not need to be asked in advance or get information (*n* = 382)	25.7% (21.3–30.1)
I am willing to share my information and do not need to be asked in advance if there is a possibility for me to leave the register (*n* = 385)	25.9.% (21.5–30.3)
I am willing to share my information but I want to be asked in advance and I want to be able to leave the register (*n* = 657)	44.3% (40.5–48.1)
I don’t want to share my information (*n* = 61)	4.1% (2.1–6.1)

Results presented as proportions with a 95% confidence interval.

Furthermore, of the respondents taking a stand on patients’ possibilities to restrict the access to their medical records by medical staff (*n* = 1614), 77.1 per cent (CI 74.8–79.4) found this possibility to be important but had not used it themselves; 2.2 per cent (CI 0.0–7.0) had used the possibility. However, 20.7 per cent (CI 16.4–25) did not think that it should be possible for patients to decide on such matters.

When asked about the importance of avoiding health data from registers and databases ending up in the wrong hands, a large majority of those responding (*n* = 1451) either completely (83.3 per cent, CI 81.4–85.2) or partly (14.3 per cent, CI 12.5–16.1) agreed that this is important. Only 2.4 per cent did not agree that this is important.

### The Weighing of Interests against One Another Throws Light on Priorities

**Figure 1.**
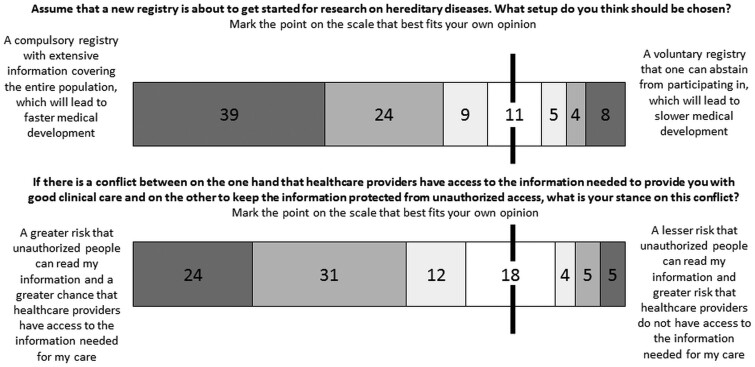
The importance of consent relative to medical progress (upper part, *n* = 1510), and the importance of avoiding unauthorized access to patient information relative to ensuring healthcare staff access to information needed (lower part, *n* = 1502). The vertical line indicates the neutral/middle part of the scale. Results presented as proportions (per cent).

In some of the survey questions, respondents were asked to weigh interests against one another. The results show that most respondents in this survey are willing to give up the demand for consent if important values, such as medical progress, are at stake (Figure 1, upper part). More precisely, 72 per cent of the respondents placed themselves on the ‘faster medical progress’ part of the scale, hence indicating that they give more weight to medical progress than to voluntariness of participation. Seventeen per cent placed themselves on the right-hand side, indicating that they give more weight to the voluntariness of participation than to speedy medical progress.

In another question where interests stood against each other, fear of unauthorized access to medical records was balanced against the risk of healthcare staff not being able to access information of importance for the care of the patient (Figure 1, lower part). Here, 67 per cent gave priority to health information being available where needed, while 14 per cent gave priority to avoid unauthorized access to their health data.

## Discussion

The main results of our study consist of three connected parts. First, our results show that the great majority of the respondents have a positive attitude towards the use of their electronic health data, both for purposes relating to their own health and for purposes primarily benefitting others. More than 9 out of 10 respondents reveal a positive attitude, regardless of whether the purpose of data use is their own care, quality improvement, research or education within healthcare. However (the second part), to be able to influence how their health data are used, by whom, and for what purpose is important for many, also among those with a positive attitude towards the use of their health data. For instance, there is a desire to be able to limit access to medical records and to avoid unauthorized access. Nevertheless (and this is the third part), when forced to prioritize, the possibility to influence the use of one’s health data is reported to be less important than e.g. medical progress by a majority of the respondents. Also, most respondents consider the risk of unauthorized access to their health data less important to avoid than the risk of healthcare staff not being able to access the information needed for their care.

That most respondents reveal a positive attitude towards the use of health data is in line with results from many other studies, including those reviewed by Skovgaard and colleagues (Skovgaard *et al.*, 2019). Articles in that review focused on the reuse of data for a number of purposes, such as research, quality assurance and planning and policy purposes. Our study also included the use of health data for the patient’s own benefit.

Some of our results are of particular interest when it comes to policymaking in the area of health data. Policymaking, however, is fundamentally based on normative deliberations. Before discussing our empirical results, a few words will therefore be said about why empirical findings may be relevant for normative conclusions.

### The Relevance of Empirical Input to Normative Conclusions

The idea behind studies doing empirical work relating to normative issues is that the empirical input indeed has some relevance to the issues at hand. As it may not be entirely clear how this could be, it is worth briefly repeating some well-established points on the matter. First, you cannot draw normative conclusions directly from empirical input (there is no direct step from *is* to *ought*)—for instance, from the fact that certain companies would love to dump their toxic waste directly into the ocean, it does not follow that they should be allowed to. Or, closer to the theme of the present paper, the fact that people have views and preferences regarding the handling of their own personal data does not mean that they thereby provide the answer to what should be done. Hence, surveys and interview studies cannot replace normative reflection and judgment when it comes to making policy decisions. However, empirical input can still be relevant to normative argumentation. From the combination of empirical input and at least one normative premise, a normative conclusion can be drawn. For instance, for those holding that autonomy and privacy are normatively relevant aspects to consider, the autonomous views of individuals become relevant to normative argumentation, although not by themselves conclusive; there may be other ethical considerations as well in the case at hand that carry more normative weight. Furthermore, all else being equal, policies matching the attitudes of the public are more likely to be perceived as legitimate. This in turn may influence behavior and, hence, the outcome—in such a case, perceptions would be of relevance to consequentialist considerations. Keeping this in mind, we will now discuss our most important empirical findings.

### Control, Consent Options and Their Costs to Medical Evaluations and Research

The desire to be in control of one’s health data is commonly reported in the international literature (e.g. Kass *et al.*, 2003; Damschroder *et al.*, 2007; Institute of Medicine, 2009). As our results show, many respondents wish their data to be used on the condition that they get to influence that use, although individuals prefer to exercise control in different ways. For example, while around 70 per cent of the respondents expressed a positive attitude towards health data being entered into registries for research given some possibility to exercise control, around two-thirds of these preferred to be asked beforehand while one third were satisfied with receiving information and being able to leave the register (Table 2). These findings are in line with those of previous studies (e.g. Rothstein 2009). For example, Willison *et al.* asked about consent choices for using health data for quality improvement and found that 20 per cent of respondents preferred the ‘notice with opt-out’ option, while some 45 per cent preferred more traditional consent procedures (Willison *et al.* 2009).

Being granted control over one’s health data can be considered a matter of principle stemming from ideas of privacy and personal autonomy. A common feature of definitions of privacy, in particular informational privacy, concerns individuals’ opportunities to affect who knows what about them (DeCew, 2018). Privacy understood this way is clearly linked with personal autonomy—the right of the individual to decide on matters particularly concerning him- or herself—which is typically aimed to be respected in the healthcare setting and in medical research (Beauchamp and Childress, 2019). The common requirement of obtaining informed consent from the patient before providing treatment stems from this respect for autonomy.

However, informed consent procedures as normally applied in health care and research provide a limited kind of influence. Patients/participants are usually faced with a choice of accepting a certain procedure or not, or deciding among a limited set of options, but are very rarely invited to negotiate the options or able to initiate the exchange. This is the case simply because those things are already settled when consent is asked for. For instance, people are asked to consent to participate in a study when all details about the study, including data management, are already decided. And a hospital’s data management system cannot be rebuilt every time a new patient has a novel preference. But considering the normative foundations of autonomy and privacy, it could be argued that, in order to better cater to the varied preferences of the public, individuals should be offered not only the choice of whether or not to have their data used but also of how to exercise influence. For instance, there might be opportunities for interaction among patients or patient representatives and hospital managers before new systems are put into place, and when modifications are discussed. And for some kinds of research at least, an interaction beforehand with concerned patient groups might open up for adjustments when it comes to what participation options are later offered. For example, the patient could be offered the option to provide either broad or narrow consent and to decide for how long a given consent is valid. In the literature, these matters have been discussed extensively (Buckley *et al.*, 2011; Helgesson, 2012; Taylor and Taylor, 2014; Eloranta and Auvinen, 2015). It has also been discussed, as a way to allow patients to exert influence, that they should have the right to donate their data to whomever they want, to be used for any purpose, for example by publishing them online (Westman, 2019). Currently, that is typically not possible due to legal constraints stemming from paternalistic concerns for the individual. If we are serious about the right to personal autonomy, such limitations may be difficult to defend.

There are, however, also arguments against letting people influence the use of their health data (see Belfrage, 2011). Arguments specifically against requiring that informed consent is obtained include that doing so generates negative consequences in the form of unreasonably high costs for some kinds of studies and leads to selection bias, hence slower progress or distorted results when it comes to medical evaluations or research (Taylor, 2008; Rothstein, 2009; Belfrage, 2011). This means that even if there may be gains from giving patients and research participants (greater) influence over the use of their data, there are also costs tied to this.

When respondents in our study were presented with a choice between on the one hand fast medical progress and compulsory inclusion of one’s data in health registers and on the other slower medical progress and voluntary inclusion of one’s data in health registers, a large majority (72 per cent) gave priority to medical progress rather than individual control of data use (Figure 1). This could be interpreted as saying that when they made the overall decision on how to balance the different ethically relevant aspects, their conclusion was that individual control of data used was of comparably lesser importance. This suggests what their priorities are, but it does not follow that these priorities should be enacted as policy. Besides, it is not obvious that voluntariness is necessarily a hindrance to medical progress or cause skewed results—these are empirical claims that warrant further investigation (as suggested by Rothstein, 2009).

### Data Protection as a Means to Avoid Harm

The desire to influence the use of one’s health data may at least partly be explained by the desire of individuals to avoid harmful consequences for themselves of sensitive personal information ending up where it should not or being used for purposes they do not support (cf. Rothstein, 2009). In other words, people may wish to avoid social, economic, and other harms that may result from actors using the data for questionable purposes, such as spreading gossip, discriminating on the labor market or direct scams adapted to the healthcare needs of the individual (e.g. Sunstein, 2001). The identification of a number of potential harms following from others’ misuse of sensitive personal information indicates the importance of data protection since proper data protection can be a means to avoid or reduce these harms. Previous studies have shown that trust in the secure handling of data is essential for people’s willingness to participate in research (e.g. Damschroder *et al.*, 2007). For instance, in a study in the USA, the authors concluded that there is considerable willingness to allow the use of patient data in research, on the condition that secure data handling is granted (Kass *et al.* 2003). This means that if participation is valuable, there are two gains from proper data protection: avoidance of harm and greater willingness to participate.

In response to a choice between a greater risk that unauthorized people can access one’s health information and a greater risk that healthcare staff cannot access health information needed for providing care (Figure 1), the majority preferred the former. This should not come as a surprise, considering what is at stake in such a situation: one’s own health and perhaps even one’s life. Some patients worry that they may fare badly or even die if healthcare staff cannot access information about their diagnoses and medication (Vårdanalys, 2017). However, when thinking about how to apply this more generally to the healthcare context, we should ask whether such choices really have to be made. It seems reasonable to aim at both avoiding unauthorized access and ensuring that data is available where and when it should.

### Policy Implications

As noted above, there are several difficulties related to using empirical input to support policy decisions. First, there is no direct step to be taken from input on what is to conclusions on what ought to be. For example, if we consider each individual to have a right to control their data, stemming from the right to have one’s autonomy respected, then this right must not be disregarded on the basis of empirical results showing that a majority of respondents are willing to waive that right.

Second, there are often difficulties interpreting empirical information in order to be clear about how it informs policy considerations. For instance, responses may be highly sensitive to the exact phrasing of claims and questions, so that minor adjustments greatly influence responses (cf Nass *et al.*, 2009). This is arguably more likely to be the case when the area described is complex and contains many variables. In the survey question presenting respondents with a dilemma, the benefits of a compulsory register were described as more specific and certain (faster medical progress, research on a hereditary disease) than what may often be the case in real life when health data is to be included in registers. Questions of this kind are important as there are often trade-offs to be made in real life between e.g. the protection of privacy and the benefits to be obtained from extensive use of data. Views on where to strike the balance are therefore of relevance for policymaking. However, it cannot be excluded that a somewhat different phrasing of the question, for instance, one slightly playing down the certainty of progress, would have produced a different response—or that people’s priorities in fact are quite fine-grained, for instance, when it comes to balancing access and usefulness on the one hand and data protection and individual influence over one’s health data on the other. According to Gandy, answers to trade-off questions are often given exaggerated interpretations which are used to legitimate policy decisions beyond what is reasonable (Gandy, 2003). In order not to make mistakes of that kind and to dig deeper into the perceptions of the relative importance of these different aspects, a large set of fairly fine-grained comparative questions would be needed.

Third, it can be debated, and needs to be considered in each case, whether the information on, say, attitudes or fears should be taken at face value or if there are reasons to ponder whether the attitudes or fears would change if the persons concerned would be better informed.

With these warning signs raised, there are nevertheless some policy conclusions to draw from our study: proper data protection is perceived by many as important and influences patients’ willingness to have their health data stored and used for various purposes. This clearly implies that data protection should be taken very seriously. However, since also availability is seen as highly important, and lack of availability may reduce the ability of health care to help patients, we should strive for data handling systems that can grant both proper data protection and good availability.

Since our data suggest that people want to exercise influence over the use of their health data, there is a need to search for solutions that reduce the potential conflict between, for instance, informed consent procedures and research quality. However, it also needs to be further explored if the willingness to be in control of one’s data mainly relates to fears of being harmed or to exercising one’s autonomy. If the former, then improved data protection and a constructive open debate on good research practices and threats to research quality might further increase acceptance for allowing some kinds of data uses in research without informed consent. Perhaps public influence over research practices can take other forms that are more satisfying to patients and the general public.

An important issue avoided so far is the relevance of context, including cultural context, to policy. This study concerns responses from the Swedish population. It could therefore be discussed whether the results are relevant also beyond the Swedish context. As mentioned in the Introduction, some aspects about Sweden are worth pointing out: the Swedish population is known to have a general high level of trust in Swedish authorities, in the healthcare system, and in research (Martinsson and Andersson, 2020), Sweden has a long tradition of registries covering a large proportion of the population, and furthermore, as digitalization has reached far in many sectors of society, including everyday life, people are familiar with and can imagine both benefits and risks generated by increased processing of electronic data. A recent Eurobarometer can be interpreted as illustrating the understanding among the population of potential benefits of data sharing as well as a high level of trust: 82 per cent of the Swedish respondents were willing to share their personal data for improving medical research and care, to be compared with the European average of 40 per cent (European Commission, 2019). Sweden, in other words, differs from other countries in important ways, which means that Swedish experiences cannot be directly extrapolated to other contexts.

However, some general things can nevertheless be learnt. First, countries facing low levels of trust need to work on their trustworthiness in order to achieve strong public support for the use of health data. They also need to engage in open debate on what data management systems health care should use, and the cost of distrust, in order to seek public support for a system that makes at least the most essential data access acceptable to patients. When it comes to research on health data, trust is essential. If people do not trust research, they will not accept to participate. If their data are used in research without their permission, that is likely to have political repercussions that are potentially harmful for research in the long run. Second, it must be borne in mind that also people with a positive attitude towards sharing of health data may want to influence how their data is used.

## Strengths and Weaknesses

The main strength of this study is that the survey questions bring up attitudes to access and control of electronic health data from a number of angles, including ones involving priority issues. It is one thing to state whether something, such as informed consent, is important or not—and quite another to take a stand on its importance relative to other things of value, such as scientific progress. Therefore, it is informative to explore how people prioritize between things they value, as this study makes possible. Some of the most interesting results in this study—on the relative importance of the control of data use and of avoiding unauthorized access to one’s data—stem from such questions. However, questions requiring prioritization may also be more difficult to answer. The dropout rate for the two questions involving priorities (8 per cent and 9 per cent, respectively) was substantially higher than for other more straightforward questions, which may be an indication of this. The dropout rate might also reflect an unwillingness to accept the conditions of the prioritization questions, for instance, that a compulsory health register is more conducive to medical development than a voluntary register, which is suggested in one of our questions.

The survey was answered by a fairly large number of individuals (*n* = 1645), but the response rate was low (31 per cent), which is the main weakness of the study as it opens up for selection bias. We suggest the following possible explanations for the low response rate: first, it is an expected consequence of the stratification procedure (including more potential respondents from subgroups with an expected low response rate in order to achieve enough responses from those groups); second, there has been a general decline in response rates to research surveys during the last decades; third, the questionnaire was sent out during the winter holidays—a period often thought of as unsuitable for surveys; and fourth and last, the topic of the survey may be considered difficult, impeding some from answering. Even though the response rate was low, respondents seem to be broadly representative of the Swedish population when it comes to known demographic variables (age, gender, education), and similar to respondents in other studies when it comes to self-estimated health and trust in authorities (Vårdanalys, 2017). On the other hand, respondents may not be representative of the population when it comes to the attitudes to the use of electronic health data—the topic of investigation. It may be suspected that those answering the questionnaire care more about the issues investigated than people in general—but it is not obvious that their opinions about how data should and should not be used are different from those of non-responders.

## Conclusions

Our results show that the great majority of the respondents have a positive attitude towards the use of their electronic health data, both for purposes relating to their own health and for purposes primarily benefitting others, but also that they want to be able to influence the use of their personal health data and have it protected from unauthorized access. Nevertheless, when asked to prioritize, the possibility to influence the use of one’s health data was reported to be less important than e.g. medical progress by a majority of the respondents, and accessibility for one’s own treatment was reported to be more important than limiting access to medical records and avoiding unauthorized access.

Our empirical results point to the need to resolve or reduce conflicting interests between, on the one hand, proper data protection and availability and, on the other, informed consent procedures (meant to assure respect autonomy and privacy) and research quality. There is also a need to further explore how people balance these interests when they unavoidably conflict.

## Supplementary Material

Supplementary material is available at *Public Health Ethics* online.

## Funding

This work was supported by the Swedish Foundation for Humanities and the Social Sciences [RMP14_1599 to S.B.].

## Supplementary Material

phaa035_Supplementary_Appendix
