# Supplementary material for: Willingness to Share yet Maintain Influence: A Cross-Sectional Study on Attitudes in Sweden to the Use of Electronic Health Data
Source: Public Health Ethics. 2020 Nov 27;14(1):23–34. doi: 10.1093/phe/phaa035 (PMC8254641; doi:10.1093/phe/phaa035)
Supplement: phaa035_Supplementary_Appendix [file phaa035_supplementary_appendix.docx]

## Appendix: Survey questionnaires used in this study

1. Would you say that you are mostly in favour of or mostly against that different healthcare units have access to each other’s medical records?

- I am mostly in favour
- I am mostly against
- I cannot take a stand

1. As a patient you may control and restrict access to your medical record. What do you think about that opportunity, and have you used it yourself?

- I have used the opportunity to restrict access to my medical record
- I have not used that opportunity, but it is important that it exists
- I don’t think that it should be possible for patients to decide on this

1. What do you think about the following statements?

Generally, authorized staff should be allowed to use information in medical records for:

1. *Medical follow up of the quality of healthcare?*
2. *Certain research?*
3. *Education within healthcare?*

- No, never
- Yes, but only with patient consent
- Yes, even without patient consent
- I don’t know/I don’t have an opinion

1. Information about your and other people’s care and health can be of much use for research and in order to learn about what treatments have the best effect. Much information is for instance collected in national registers for specific diagnoses in order to facilitate follow up and quality improvement of care. What is your view on information about you being entered into such registers?

- I am willing to share my information and don’t need to be asked in advance or get information
- I am willing to share my information without being asked in advance if there is an opportunity for me to leave the register
- I am willing to share my information, but I want to be asked in advance and I want to be able to leave the register
- I don’t want to share my information
- I don’t know

1. How well do the following statements reflect your view concerning registers and databases on healthcare and health?

“It is important for me that data does not end up in the wrong hands”

- This reflects my view completely
- This reflects my view rather well
- This reflects my view rather poorly
- This does not at all reflect my view
- I don’t know/I don’t have a view

1. Assume that a new registry is about to get started for research on hereditary diseases. What setup do you think should be chosen?

Mark the point on the scale that best fits your own opinion

3 2 1 0 1 2 3

A compulsory register with A voluntary register

extensive information cover- that one can abstain

ing the entire population, from participating in,

which will lead to faster which will lead to slower

medical development medical development

1. If there is a conflict between on the one hand that healthcare providers have access to the information needed to provide you with good clinical care and on the other to keep the information protected from unauthorized access, what is your stance on this conflict?

Mark the point on the scale that best fits your own opinion

3 2 1 0 1 2 3

A greater risk that A lesser risk that

unauthorized people unauthorized people

can read my information can read my information

and a greater chance that and a greater risk that

healthcare providers healthcare providers

have access to the will not have access to the

information needed for information needed for

my care my care
